# Inhospital cardiac arrest — the crucial first 5 min: a simulation study

**DOI:** 10.1186/s41077-022-00225-0

**Published:** 2022-09-09

**Authors:** Mathilde Stærk, Kasper G. Lauridsen, Camilla Thomsen Støtt, Dung Nguyen Riis, Bo Løfgren, Kristian Krogh

**Affiliations:** 1 Department of Medicine, Randers Regional Hospital, Randers, Denmark; 2 Education and Research, Randers Regional Hospital, Randers, Denmark; 3 Department of Emergency Medicine, Gødstrup Hospital, Herning, Denmark; 4 Research Center for Emergency Medicine, Aarhus University Hospital, Aarhus, Denmark; 5 Emergency Department, Randers Regional Hospital, Randers, Denmark; 6 Department of Anesthesiology and Critical Care Medicine, Children’s Hospital of Philadelphia, Philadelphia, USA; 7 Department of Emergency Medicine, Regional Hospital Horsens, Horsens, Denmark; 8 Department of Clinical Medicine, Aarhus University, Aarhus, Denmark; 9 Department of Anaesthesiology and Intensive Care, Aarhus University Hospital, Aarhus, Denmark

**Keywords:** Inhospital cardiac arrest, Basic life support, In situ simulation

## Abstract

**Background:**

Early recognition and call for help, fast initiation of chest compressions, and early defibrillation are key elements to improve survival after cardiac arrest but are often not achieved. We aimed to investigate what occurs during the initial treatment of unannounced in situ simulated inhospital cardiac arrests and reasons for successful or inadequate initial resuscitation efforts.

**Methods:**

We conducted unannounced full-scale in situ simulated inhospital cardiac arrest followed by a debriefing. Simulations and debriefings were video recorded for subsequent analysis. We analyzed quantitative data on actions performed and time measurements to key actions from simulations and qualitative data from transcribed debriefings.

**Results:**

We conducted 36 simulations. Time to diagnosis of cardiac arrest was 37 (27; 55) s. Time to first chest compression from diagnosis of cardiac arrest was 37 (18; 74) s, time to calling the cardiac arrest team was 144 (71; 180) s, and time to first shock was 221 (181; 301) s. We observed participants perform several actions after diagnosing the cardiac arrest and before initiating chest compressions. Domains emerging from the debriefings were *teaming* and *resources*. *Teaming* included the themes *communication*, *role allocation*, *leadership*, and *shared knowledge*, which all included facilitators and barriers. *Resources* included the themes *knowledge*, *technical issues*, and *organizational resources*, of which all included barriers, and *knowledge* also included facilitators.

**Conclusion:**

Using unannounced in situ simulated cardiac arrests, we found that key elements such as chest compressions, calling the cardiac arrest team, and defibrillation were delayed. Perceived barriers to resuscitation performance were leadership and teaming, whereas experience, clear leadership, and recent training were perceived as important facilitators for treatment progress.

## Background

Survival after inhospital cardiac arrest (IHCA) is low at approximately 20–30% [1–4]. The international resuscitation guidelines emphasize the key elements in the “chain of survival,” i.e., early recognition and alert of emergency services, early initiation of chest compressions, and early defibrillation [5–7]. The chance of survival decreases with delays in any or all of these key components [7–10]. While previous studies report that guideline adherence improves the chance of survival, studies also showed limited guideline adherence and delays in initiation of treatment [11–13].

Despite this, several studies have focused on investigating the advanced life support after the arrival of a cardiac arrest team or investigated the quality of chest compressions [14–18]. In contrast, what occurs during the important first few minutes in a clinical setting has received less attention. Due to the unpredictability of IHCAs, it is challenging to investigate the first minutes of a clinical cardiac arrest. However, unannounced in situ simulation is a method to create the opportunity to explore what happens during the initial response to a cardiac arrest. In situ simulations have previously been used to test the emergency response at hospitals and revealed many patient safety threats such as missing or malfunctioning equipment and lack of knowledge [19–23]. Furthermore, in situ simulations have also been shown to improve patient outcomes, teamwork, self-confidence, and decrease time to key elements within resuscitation [24–32].

Accordingly, using a mixed-method approach, we aimed to investigate what occurs during the initial treatment of unannounced in situ simulated IHCAs and explore the perceived reasons for successful or inadequate initial resuscitation efforts from debriefings.

## Material and methods

### Study design

This mixed-method study is a post hoc analysis of a prospective, multicenter, observational, simulation study [33]. Full-scale unannounced in situ simulated cardiac arrests were conducted in different hospital departments and were followed by a debriefing. We present data on the treatment before the cardiac arrest team’s arrival.

### Setting

Simulations were conducted during the day shift (9.00–15.00) and the night shift (16.00–00.00) on regular workdays and weekends. Participants were the ward staff (i.e., physicians, nurses, and nurse assistants) and the responding cardiac arrest team to reflect clinical practice. Hospital departments were eligible if the department had access to an automated external defibrillator (AED). Intensive care, cardiology, psychiatric, and pediatric departments were excluded as they had different procedures and/or competences to treat a cardiac arrest and as we only conducted scenarios with adults.

On the day of the simulation, the nurse manager assigned a patient room for the simulation without informing other staff members. Time, location, and scenario were unknown to all other staff. A full-body manikin (Resusci Anne QCPR AED with Airway Head, Laerdal Medical, Stavanger, Norway), dressed in patient garments and with an intravenous access, was placed in a hospital bed. The manikin collected data on the quality of cardiopulmonary resuscitation. Before and during the study period, all hospital staff with the possibility of being involved in a simulation received a written notification to act as in a real situation in case of a simulation. After the simulation and debriefing, participants were sent an e-mail with a questionnaire inquiring demographic information.

### Scenario

A nurse/nurse assistant was called to the patient room and briefed with a short patient story: female patient admitted to observation for (illness related to the department), not severely affected but now experiencing chest pain and has called for help*.* The nurse/nurse assistant was instructed to assess the patient and to act as in a real situation throughout the entire simulation.

The manikin was unconscious with no breathing. The manikin presented with ventricular fibrillation when connected to a defibrillator until the cardiac arrest team had delivered two shocks. At subsequent rhythm analysis, the manikin presented with sinus rhythm. The simulation ended either when sinus rhythm was discovered or 3 min after the rhythm analysis with sinus rhythm.

It was possible to retrieve a patient record with a history, standard tests including blood pressure, saturation, blood samples, an electrocardiogram, and chest X-ray. Participants could perform actions as in a real situation, e.g., administration of intravenous medication, and intubation. Participants had to retrieve and use all their normal equipment. ShockLink (Laerdal Medical, Stavanger, Norway) was connected to the defibrillator by a research assistant to allow for defibrillation. Participants received no help nor feedback during simulations except if participants performed an action correctly and asked for the result, e.g., if they checked for breathing, an answer was given, i.e., the patient is not breathing.

### Debriefing

We conducted a semi-structured debriefing of 15–25 min for all participants at each simulation. The debriefing guide was based on PEARLS [34, 35]. During the debriefing, we used plus-delta in the analysis phase [36] and strived to use advocacy inquiry [37] when suited. The debriefings were conducted openly without fixed topics to allow for participants’ emerging themes and discussion points.

### Ethics

The Regional Committee on Biomedical Research Ethics waived the need for permission and deemed the study exempt from individual consent (141/2017). The study was approved by the Danish Data Protection Agency (1-16-02-367-18). Permission to conduct the study was granted from all hospital administrations. All participants were given written information prior to the study period as well as regularly throughout the study period. Participants were informed that participation was voluntary, that data would be de-identified after analysis, and no information on individual performance would be disclosed. Furthermore, they were informed that refrainment from participating would not be disclosed to the management. Safety precautions were taken to ensure patient safety during simulations, and in case of emergencies/acute patients, simulations were canceled/interrupted [33].

### Data collection

The in situ simulations and debriefings were video recorded. Two cameras (GoPro Hero 5 Black, San Mateo, CA, USA) were placed in opposite corners of the room and captured 180-degree video. An additional camera was used to ensure sound quality during debriefings. Data regarding actions performed during the simulation and time measurements were obtained from the video recordings by two independent researchers. Data on the quality of cardiopulmonary resuscitation were retrieved from a SimPad (Laerdal Medical, Stavanger, Norway) wirelessly connected to the manikin. Time zero was considered as the time of diagnosis of cardiac arrest unless otherwise stated. The study was reported in accordance with the STROBE guidelines.

### Outcome measures

Outcomes were (A) time to diagnosis of cardiac arrest, (B) time to first chest compression, (C) time to first shock, (D) chest compression fraction, (E) actions performed during delays of other actions, and (F) participants’ motivation for initial actions.

### Qualitative analysis

Video recordings of the debriefings were transcribed verbatim and analyzed using a qualitative approach [38]. Three researchers (MS, KGL, KK) independently coded six debriefings inductively to develop a coding framework. A single researcher (MS) coded the remaining debriefings. Furthermore, three debriefings were coded by the same three researchers to ensure continued agreement halfway through. Codes were merged into themes for interpretative thematic analysis [39, 40]. The three researchers (MS, KGL, KK) who performed the analysis are all medical doctors, resuscitation researchers, and simulation instructors. All had experience with using thematic analysis.

### Quantitative analyses

Categorical data are presented as percentages (number), normally distributed data are presented as mean (standard deviation), and non-normally distributed data are presented as median (1st quartile; 3rd quartile). Histograms and QQ plots determined normality. To see if there were any differences in actions performed in courses of treatment that were effective and those that were ineffective, we compared the fastest third and slowest third of simulations. Data were analyzed using R-statistics (version 1.4.1717, R Core Team 2021, R Foundation for Statistical Computing, Vienna, Austria). We did not perform a sample size calculation but intended to include 60 in situ simulations.

## Results

From July 2018 to December 2020, we conducted 36 in situ simulations (30 complete simulations and 6 interrupted simulations with partial datasets). Furthermore, 30 simulations were canceled (8 due to the COVID-19 pandemic, 7 due to lack of department capacity, 12 due to acute patients, and 3 due to other reasons). The data collection was stopped early due to the COVID-19 pandemic. The simulations included 249 ward staff members. Participant demographics are shown in Table 1.

**Table 1:** Demographics

Gender, female^a^	93% (*n* = 129)
Age, years^b^	32 (28; 46)
Profession	
Physician	14% (*n* = 21)
Nurse	68% (*n* = 102)
Orderly	3% (*n* = 4)
Other	15% (*n* = 22)
Years of experience^c^	4 (1; 11)
Time since last resuscitation training	
Within 6 months	30% (*n* = 44)
6–12 months	23% (*n* = 34)
1–2 years	35% (*n* = 52)
2–3 years	7% (*n* = 11)
More than 3 years	5% (*n* = 8)

Demographics are available for 149 of 249 ward staff members. Data presented as percentages (*n*) or median (Q1; Q3). ^a^Data missing for 10 participants, ^b^data missing for 8 participants, ^c^data missing for 27 participants

### Quantitative data

In all 36 in situ simulations, the ward staff initiated basic life support. Cardiac arrest was correctly diagnosed in 67% (*n* = 24) of simulations according to the European Resuscitation Council Guidelines [5, 41]. In 28% (*n* = 10) of simulations, participants only examined for unconsciousness, and in 6% (*n* = 2) of simulations, participants only checked for breathing. Of the 26 simulations where breathing was examined, participants opened the airway in 15% (*n* = 4) of simulations. Participants performed various actions prior to diagnosing the cardiac arrest (Table 2) as well as prior to initiating chest compressions (Table 3).

**Table 2:** Actions performed from the beginning of simulation until diagnosis of cardiac arrest

	All simulations	Fastest group 20 (16; 26) s	Slowest group 78 (60; 118) s
**Always necessary**			
Talks loudly to manikin	94% (*n* = 34)	92% (*n* = 11)	92% (*n* = 11)
Shakes manikin	83% (*n* = 30)	83% (*n* = 10)	75% (*n* = 9)
Checks for breathing	67% (*n* = 24)	92% (*n* = 11)	58% (*n* = 7)
**Sometimes necessary**			
Removes bed rail(s)	58% (*n* = 21)	50% (*n* = 6)	33% (*n* = 4)
Activates internal alarm	36% (*n* = 13)	33% (*n* = 4)	33% (*n* = 4)
**Never necessary**			
Prepares to measure blood pressure and/or saturation	11% (*n* = 4)	0	33% (*n* = 4)
Pain stimulates manikin	8% (*n* = 3)	8% (*n* = 1)	8% (*n* = 1)
Calls doctor	8% (*n* = 3)	0	8% (*n* = 1)
Gives verbal handover to colleagues without performing other actions	8% (*n* = 3)	0	17% (*n* = 2)
Removes duvet	8% (*n* = 3)	25% (*n* = 3)	0
Exposes manikin’s chest	8% (*n* = 3)	8% (*n* = 1)	8% (*n* = 1)
Checks for pulse	6% (*n* = 2)	0	8% (*n* = 1)
Leaves the room to get help	6% (*n* = 2)	8% (*n* = 1)	0
Moves the bed	6% (*n* = 2)	0	17% (*n* = 2)
Removes pillow	6% (*n* = 2)	0	8% (*n* = 1)
Raises the bed	3% (*n* = 1)	0	8% (*n* = 1)
Collects equipment	3% (*n* = 1)	0	8% (*n* = 1)

Data presented as median (Q1; Q3) or percentages (*n*). The fastest group included the 1/3 of simulation with the fastest time to diagnose cardiac arrest, whereas the slowest group included the 1/3 of simulation with the longest time to diagnose cardiac arrest

**Table 3:** Actions performed from diagnosis of cardiac arrest until initiation of chest compressions

	All simulations	Fastest group 13 (10; 17) s	Slowest group 93 (79; 108) s
**Always necessary**			
Activates internal alarm	56% (*n* = 20)	42% (*n* = 5)	33% (*n* = 4)
**Sometimes necessary**			
Exposes manikin’s chest	42% (*n* = 15)	8% (*n* = 1)	67% (*n* = 8)
Removes bed rail(s)	28% (*n* = 10)	17% (*n* = 2)	25% (*n* = 3)
Removes duvet	25% (*n* = 9)	8% (*n* = 1)	33% (*n* = 4)
Removes pillow	19% (*n* = 7)	0	25% (*n* = 3)
Attaches automated external defibrillator	3% (*n* = 1)	0	8% (*n* = 1)
**Never necessary**			
Lowers bed	44% (*n* = 16)	42% (*n* = 5)	33% (*n* = 4)
Talks loudly to manikin	28% (*n* = 10)	17% (*n* = 2)	25% (*n* = 3)
Moves the bed	25% (*n* = 9)	8% (*n* = 1)	50% (*n* = 6)
Shakes manikin	22% (*n* = 8)	8% (*n* = 1)	25% (*n* = 3)
Calls cardiac arrest team	22% (*n* = 8)	25% (*n* = 3)	25% (*n* = 3)
Collects equipment	17% (*n* = 6)	8% (*n* = 1)	25% (*n* = 3)
Removes headboard	17% (*n* = 6)	8% (*n* = 1)	17% (*n* = 2)
Delegates tasks without performing other actions	14% (*n* = 5)	0	17% (*n* = 2)
Checks for breathing	14% (*n* = 5)	17% (*n* = 2)	8% (*n* = 1)
Leaves the room to get help	14% (*n* = 5)	8% (*n* = 1)	25% (*n* = 3)
Head tilt/chin lift without checking for breathing	8% (*n* = 3)	8% (*n* = 1)	17% (*n* = 2)
Calls doctor	8% (*n* = 3)	8% (*n* = 1)	8% (*n* = 1)
Gives verbal handover to colleagues without performing other actions	8% (*n* = 3)	8% (*n* = 1)	8% (*n* = 1)
Checks for pulse	6% (*n* = 2)	0	8% (*n* = 1)
Checks for foreign body in airways	6% (*n* = 2)	0	17% (*n* = 2)
Prepares ventilation equipment	6% (*n* = 2)	0	17% (*n* = 2)
Reads patient record	3% (*n* = 1)	8% (*n* = 1)	0
Performs bag-mask ventilation	3% (*n* = 1)	0	8% (*n* = 1)
Raises the bed	3% (*n* = 1)	0	8% (*n* = 1)

Data presented as median (Q1; Q3) or percentages (*n*). The fastest group included the 1/3 of simulation with the fastest time to diagnose cardiac arrest, whereas the slowest group included the 1/3 of simulation with the longest time to diagnose cardiac arrest

**Fig. 1:**
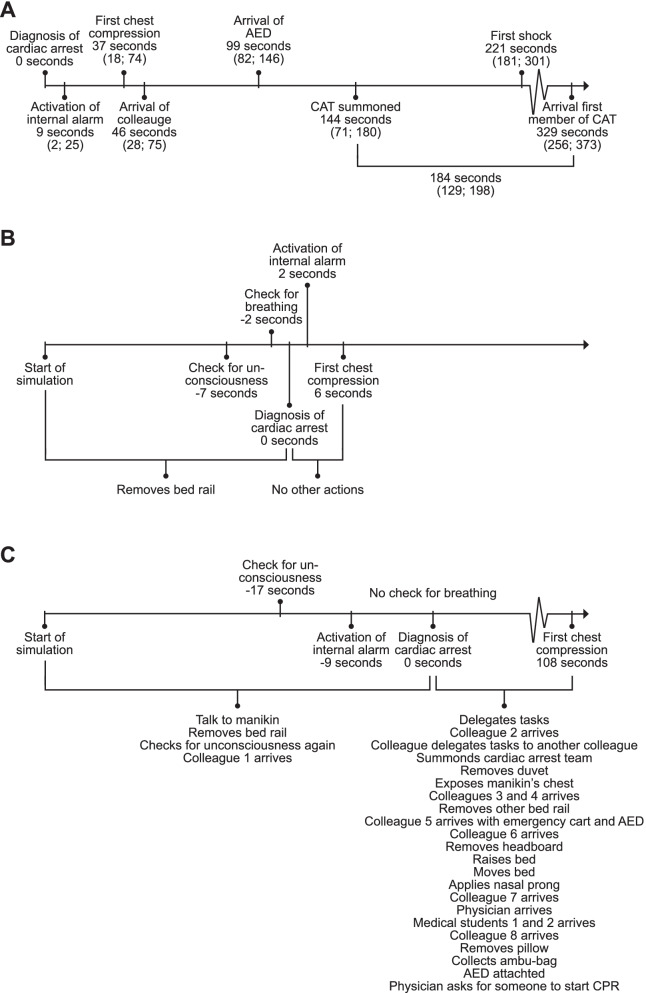
Timelines. **A** Median times for actions. Data presented as median (Q1; Q3). **B** Example of an effective timeline. **C** Example of an ineffective timeline. AED, automated external defibrillator; CAT, cardiac arrest team; CPR, cardiopulmonary resuscitation

Time from start of the simulation to diagnosis of cardiac arrest was 37 (27; 55) s. Time to first chest compression was 37 (18; 74) s from diagnosis of cardiac arrest, time to calling the cardiac arrest team was 144 (71; 180) s, and time to first shock was 221 (181; 301) s from diagnosis of cardiac arrest. Other time measurements and examples of actions performed are presented in Fig. 1. The mean chest compression depth was 47 (9) mm (recommendation 50–60 mm [5]), mean chest compression rate was 105 (15) compressions per minute (recommendation 100–120 per minute [5]), and mean chest compression fraction was 0.73 (0.15).

### Qualitative data

From the debriefings, we identified two domains related to the initial treatment of the simulated cardiac arrests: (A) *teaming* and (B) *resources*. The domain *teaming* (ad hoc formation of teams and teamwork [42]) consisted of four themes: *communication*, *role allocation*, *leadership*, and *shared knowledge*, which all included barriers and facilitators. The domain *resources* consisted of three themes: *knowledge*, *technical issues*, and *organizational resources.* *Knowledge* included both barriers and facilitators, whereas *technical issues* and *organizational resources* themes were only brought up in the debriefings when they were barriers.

Within *teaming*, it was described how *communication*, *role allocation*, *leadership*, and *shared knowledge* affected the ad hoc formation of the team and the teamwork (Table 4). Clear and audible communication had an overall facilitating effect. Closed-loop communication was often mentioned as a facilitator, whereas lack of communication was a barrier. When it came to *role allocation*, it was described how an effective role allocation contributed as a facilitator to treatment progress. Role allocation was facilitated by explicit verbalization and when the leadership role was undertaken early in the scenario. Furthermore, initial leadership and task allocation by a nurse or nurse assistant was perceived to optimize the treatment. In contrast, lack of early leadership resulted in confusion about which tasks to be addressed and who would address them. Those participants who arrived later in the scenario found it difficult to determine their role when there was no leader assigning tasks and overseeing the entire situation. Participants frequently stated it was challenging to assume a leadership position when they perceived that they lacked clinical experience/experience with cardiac arrest. Finally, it was described how assumptions about what had been done by other team members led to the omission of important tasks such as calling the cardiac arrest team. In contrast, summaries, verbalization of actions, and general audible and clear communication were facilitators contributing to shared knowledge (Table 4).

**Table 4:** Themes related to *teaming*

Theme	Facilitator	Barrier
*Communication*	Clear and audible communication, summaries, and closed-loop communication facilitated the treatment, whereas lack of these was a barrier	
	*It just helps to look at [name radiographer student] and say, “You (pointing at a colleague), run! There’s a cardiac arrest”. It’s a clear command. Instead of just shouting it out in the room, I've always been told that, then say, “YOU, run.”* (Radiographer 1-B4) *Just closed-loop [communication]. So, when [name doctor] says to [name nurse assistant], then [name assistant] replies, “I’m calling [the cardiac arrest team]”, “I’ve called”. That worked well.* (Nurse 1-D10)	*Well, I was sort of in doubt; For example, were you [cardiac arrest team] called at all…?* (Nurse 1-D13) *If someone asks us to do it, we should have repeated it. “Who has the time?”, “I have the time” or something like that.* (Nurse 1-A3)
*Role allocation*	A clearly defined role allocation and communication on role allocation — both in case of pre-defined roles as well as the adaption to ad hoc teamwork — before the arrival of the cardiac arrest team facilitated the treatment	
	*I think we were quick to divide the roles between the nurses when we arrived.* (Nurse 1-B1) *I think we did good - helping each other. One thing is leadership, another is that we’re all curious of how we can best help each other because the team leader can’t keep an eye on everything, e.g., who needs to be replaced [at chest compressions]. I think we did well, at helping each other.* (Nurse 3-D40)	*I had a hard time figuring out what I could do when I arrived, because everybody was occupied and busy doing their thing.* (Physician 1-D38) *I did chest compressions, and all of a sudden, I was standing with the medication, and I suddenly got a new task, and I thought that’s not good, because I was circulating on chest compressions with two others.* (Nurse 4-D37) *I could have used a little more structure.* (Nurse 4-D37) *Maybe it is too much to ask, but it would have been nice if someone took the lead. Then there wouldn’t be doubt about who should do what.* (Nurse 6-D37)
*Leadership*	Clear leadership from the start and clear formal shift in case of change in leadership facilitated teamwork. In contrast, lack of or doubt about the leadership and insecurity in taking the lead was a barrier	
	[Debriefer: *How was it for you that your physician clearly stated that he/she is the team leader?*]; *It is nice and feels safe* (Nurse 2-D15); *Indeed* (Nurse assistant 1-D15); *Most definitely, it works for sure.* (Nurse 1-D15) *I think it is good to know that [name physician] is standing next to me and aren’t doing anything other than keeping the overview.* (Nurse 2-D10) *You [name, nurse 1-D40] was the leader in the beginning, and I felt completely safe about it so I could just do chest compressions and hear that you delegated tasks to people.* (Physician 1-D40); *It was you [name, nurse 1-D40] who delegated.* (Nurse 3-D40); *It sort of just burst out of me.* (Nurse 1-D40); *That worked really well.* (Nurse 3-D40)	*We definitely lacked a team leader among the nurses who could delegate, but I think we were taken by surprise...* (Nurse 1-D9) *It would have been nice – because I haven’t been involved in something like this before – so I’m insecure about taking the leadership in the situation.* (Nurse 1-D37) *I think it would have been nice if the part about who’s the team leader was said out loud, like, “Now I’m team leader.”* (Nurse 1-D14)
*Shared knowledge*	It is a facilitator to share knowledge and understanding of the progress and task allocation. Shared knowledge is achieved by clear and audible verbalization, summaries, and asking questions. It was a barrier when assumptions about what had been done and the progress of treatment were made	
	*When you started to write it [treatment progress] down, I felt there was more control of what was happening.* (Nurse 8-B2) *It is about communicating it clearly and loud so everybody can hear it.* (Orderly 2-A3)	*… I just thought that I wouldn’t have been called if not the cardiac arrest team had been called already. I just assumed that the cardiac arrest team would arrive any minute, so I started acting as leader until they arrived. I thought they were en route. I just didn’t ask about it.* (Physician 1-D18); *And I just made the exact same mistake when I arrived. I just assumed that, of course, the cardiac arrest team had been called.* (Physician 2-D18) *Well, I just assumed that everybody did what they were supposed to do.* (Physician 1-B2)

For the theme *knowledge*, it was clear that (recent) experience with resuscitation either in training sessions or clinical events was a facilitator to treatment progress. Moreover, the use of cognitive aids, e.g., a leaflet with treatment algorithms or action cards, was described as helpful tools. In contrast, lack of practical training or clinical encounters was a barrier. *Technical issues* were described to cause delays, irritation, and confusion. These issues were related to, e.g., issues activating the internal alarm at the department to alert the nearest colleagues of an emergency. Furthermore, issues with operating the elevators were described. Additionally, it was described that *organizational resources*, i.e., both too few and too many people, were a barrier to resuscitation by making teaming difficult. This was especially difficult as overcrowding and understaffing were not handled in resuscitation training where instead a fixed number of people were present, each having a specific task (Table 5).

**Table 5:** Themes related to *resources*

Theme	Facilitator	Barrier
*Knowledge*	Knowledge, experience, and cognitive aids were facilitators, while lack of these was a barrier	
	*A few days ago, we had a [similar event], I think it is the experience from being involved in a few situations that helped us.* (Nurse 2-D28) *It was good that we [two physicians] could talk about it [reversible causes]. I couldn’t remember them all, but you [physician 2-D40] had a leaflet in our pocket, which was quite good.* (Physician 1-D40); *I’m a junior doctor; of course, I have the leaflet.* (Physician 2-D40) *I think it would be nice to have an action card stating, e.g., ‘take time’. Then each person could take a card with a task. Then I would know what to do. I think that would be great.* (Midwife 1-A1)	*I had been here for a little while without doing anything. I had to first remember what it was I was supposed to do.* (Nurse student 1-D26) *I’m the ’cardiac arrest coordinator’, but the disadvantage is that I have never done it myself – I’ve always just been observing – I’m not that good in practice.* (Nurse 1-A5)
*Technical issues*	Technical challenges — especially related to the alarm procedure — were a barrier	
	-	*When I tried to activate the alarm, it didn’t go off on my pager. I couldn’t hear the alarm going off anywhere!* (Nurse 1-B5) [Alarm system did not work in the room of the simulation] *It caused some confusion when I had to go out in the hallway and shout, “hurry up”, and coordinate all that. It’s confusing, and I lost control of the situation.* (Nurse assistant 1-D29) *When the alarm was sounding on the phone, it took a while before I could hear that it was a cardiac arrest. I almost ended the call because I thought it was a false alarm.* (Nurse 3-A1)
*Organizational resources*	Lack of resources, e.g., people or lack of skills, were a barrier. Also, limited training, training not equivalent to reality, or no training was a barrier	
	-	*I didn’t think we were enough people in the beginning. I had to prioritize tasks.* (Physician 1-D19) *We are lots of new staff on tonight. Some of my colleagues don’t even have resuscitation training yet.* (Nurse 1-D13) *When we trained, we trained with just the right amount of people, and then you assume that’s what it is in a real situation. Five people: one for the AED, one to collect the equipment, one to call the cardiac arrest team, one for CPR, one to lead.* (Physician 1-D26); *But we were fifteen. Ten people were just standing here.* (Nurse 2-D26)

## Discussion

Using unannounced, full-scale in situ simulations, we identified delays diagnosing cardiac arrest, initiating chest compressions, and calling the cardiac arrest team. These delays were related to the insertion of unnecessary actions, knowledge deficits, and lacking skills within treatment algorithm and teaming.

Previous studies of inhospital basic life support have focused on the quality of chest compressions or time to first shock [17, 18, 43, 44]. We were able to study deficiencies in diagnosing cardiac arrest and calling the cardiac arrest team as well as exploring the reasons for these delays by using unannounced in situ simulations followed by a debriefing. In one-third of simulations, the diagnosis was incorrect as defined in the criteria by the European Resuscitation Council, and many unnecessary actions were performed before diagnosing the cardiac arrest [5]. Most participants checking for breathing did not open the airway. Thus, it may be argued that the number of participants correctly diagnosing cardiac arrest is even lower. Notably, studies have shown that laypersons, healthcare professionals, and even basic life support instructors struggle to diagnose cardiac arrest correctly [45, 46]. While contemporary strategies to improve basic life support training focus on chest compression skills [17, 18, 47], our findings highlight that the diagnosis of cardiac arrest should be emphasized in future training and research as this is essential to initiate an early call for help and early chest compressions.

We found delays in executing key components in resuscitation, such as initiation of chest compressions, calling the cardiac arrest team, and delivering the first shock. Notably, time to first compression and time to rhythm check are considerably worse in our study compared to previous reports from cardiac arrest registries [2, 48]. This is likely due to inaccuracies in the registries that may be prone to biased time estimates [49, 50]. Chest compressions were not initiated within the first minute after diagnosing the cardiac arrest in more than 25% of cases. While delivering the first shock depends on collecting a defibrillator, which takes time, chest compressions can and should be initiated immediately and only requires a single person to start compressing the chest. These initial actions of diagnosing cardiac arrest, calling for help, and starting compressions are known to be the most important links in the “chain of survival” in terms of improving survival outcome [7–10, 43]. Therefore, emphasis on these elements in training should be prioritized to make the greatest impact on survival [51].

Participants in this study performed several other actions before starting chest compressions, e.g., moving the bed or collecting equipment. Our qualitative data showed that participants with limited knowledge and/or experience described doubt as to which actions to perform and in which order. Many of the “unnecessary” actions performed were actions that should be performed at a later stage, e.g., moving the bed and removing the headboard to optimize the access to ventilate the patient or collecting equipment for later administration of medication. While these actions are part of the ward staff’s responsibilities, their timing and prioritization were wrong. The lack of correct task performance and prioritization may be a symptom of cognitive overload in a stressful situation, thus suggesting the need for training in prioritization and delegation of tasks to initiate basic life support in the clinical setting.

The amount and frequency of resuscitation training seem to be a key factor for performing the correct algorithm and prioritizing tasks. Our participants described resuscitation experience as a facilitator to performance. However, nurses and nurse assistants in Denmark are most often only offered a basic life support course and only every other year [52]. In contrast, studies have shown that resuscitation skills decay after as little as 3–6 months. Accordingly, evidence support the use of low-dose but high-frequency training with a focus on contextualized team training [29, 53–58].

Cognitive aids could be used to remember the correct algorithm, prioritization, and allocation of tasks. Our participants found a leaflet summarizing the algorithm useful and suggested how cognitive aids may be helpful to allocate tasks and roles. Similarly, the International Liaison Committee on Resuscitation suggests cognitive aids could be beneficial for advanced life support among healthcare professionals but recommend against the use of cognitive aids for laypeople performing basic life support as it may increase time to first compression [59]. No studies exist on cognitive aids for basic life support provided by healthcare professionals. However, the risk of increased time to first compression must be considered versus the potential benefit of avoiding delays in instances where care providers are unsure of how to proceed.

We found several barriers related to *teaming*, such as communication and leadership skills, as well as the ability to allocate specific roles and tasks effectively. The term “teaming” covers the ad hoc formation of a team, including the role allocation as well as the otherwise teamwork [42]. Not only did we observe the lack of these skills to ensure effective progress of treatment, but our participants also described how lack of communication and leadership were barriers, whereas audible and closed-loop communication, effective role allocation, and good leadership were mentioned as facilitators. The ward staff in our study were mainly trained in hospital basic life support courses, primarily focusing on the technical training in performing cardiopulmonary resuscitation without specific training of nontechnical skills. Today, there is a focus on e-learning and skill stations for basic resuscitation training [60–65]. While this may be a feasible solution to practice chest compressions or to learn some elements, e.g., how to alert the cardiac arrest team, our findings show the importance of contextualized training to allow for training in nontechnical skills and familiarization with local conditions, e.g., location of equipment, which should be combined with training in communication and leadership, and team training. This should be offered for healthcare professional basic life support providers instead of the current focus on mainly individual cardiopulmonary resuscitation skills.

Our findings suggest a necessity for more frequent training emphasizing teaming, communication, and leadership in the basic life support curriculum for healthcare providers. Today, these essential nontechnical skills are reserved for the advanced life support curriculum, neglecting the importance of how to organize a group of staff members and treatment components in an emergency before the arrival of the cardiac arrest team. The future training should be contextualized using team training with equipment equal to the clinical equipment and in a setting equivalent to the clinical setting. Furthermore, the training should focus on time-sensitive key components, e.g., emphasizing the importance of early cardiac arrest diagnosis and chest compression initiation.

### Limitations

The study was simulation based. However, the study was designed to mimic the clinical setting using full-scale, unannounced in situ simulations with the normally available equipment and staffing. Despite being unannounced, clinical staff may have detected the research team entering the patient rooms in some cases. We cannot exclude a Hawthorne effect as participants were informed that a research study was being conducted. Thus, real-life clinical performance may be worse than what we found. However, we believe such an effect is limited as participants did not know time, location, or content of the simulations. During the data collection in the period of COVID-19, it was mandatory to use surgical face masks and to disinfect hands before entering the simulation. This may have caused slight delays in the arrival of subsequent providers. However, this reflected the clinical setting in that period. The study was an observational study and not based on a sample size calculation. Our sample size was limited, and the COVID-19 pandemic caused us to stop the data collection prematurely.

## Conclusion

During unannounced in situ simulated cardiac arrests, key elements such as chest compressions, calling the cardiac arrest team, and defibrillation were delayed. Several actions were performed before these key elements. Perceived barriers to resuscitation performance were leadership and teaming, whereas experience, clear leadership, and recent training were perceived as important facilitators for treatment progress.

## Data Availability

The datasets generated and/or analyzed during the current study are not publicly available due to data protection requirements but are available from the corresponding author on reasonable request.

## Abbreviations

AED: Automated external defibrillator
CAT: Cardiac arrest team
CPR: Cardiopulmonary resuscitation
IHCA: Inhospital cardiac arrest
